# Relationship between salivary inflammatory mediators and oral dryness during chemotherapy in patients with cancer: a cohort study

**DOI:** 10.1007/s00520-026-10796-7

**Published:** 2026-05-28

**Authors:** Anna Kiyomi, Kensuke Yoshida, Nana Okamoto, Akira Kurokawa, Chie Saito, Hiroko Kanemaru, Kei Tomihara, Akitsugu Ohuchi, Munetoshi Sugiura

**Affiliations:** 1 Laboratory of Drug Safety and Risk Management, Tokyo University of Pharmacy and Life Sciences, Tokyo, Japan; 2 Oral Management Clinic for Medical Cooperation, Niigata University Medical and Dental Hospital, Niigata, Japan; 3 Division of Hospital Pharmacy, Niigata University Medical and Dental Hospital, Niigata, Japan; 4 Division of Oral and Maxillofacial Surgery, Faculty of Dentistry & Graduate School of Medical and Dental Sciences, Niigata University, Niigata, Japan

**Keywords:** Oral mucositis, Saliva, Oral dryness, Cytokines, Supportive care, Biomarkers

## Abstract

**Purpose:**

To evaluate the association between salivary inflammatory mediators and oral dryness during oral mucositis development in patients with cancer undergoing chemotherapy.

**Methods:**

This prospective cohort study (time-stratified sampling) enrolled adult patients undergoing chemotherapy or chemoradiotherapy at Niigata University Medical and Dental Hospital (November 2021–August 2023). Sample size was determined a priori from a pilot study. Oral mucosal status was graded using the World Health Organization oral toxicity scale, and oral moisture was measured (dryness < 28). Unstimulated whole saliva was analyzed for interleukins (IL)-1β, IL-6, IL-8, IL-10, IL-12p70, tumor necrosis factor (TNF), prostaglandin E2, and vascular endothelial growth factor via cytometric bead array and enzyme-linked immunosorbent assay. Clinical data included demographics, treatment, and days post-chemotherapy (0–7, 8–14, ≥ 15). TNF and IL-10 were independently associated with oral dryness; cutoffs were median levels in patients with grade 0 mucositis.

**Results:**

Among 162 patients, 56 (34.6%) had oral dryness. Dryness was associated with higher neutrophil counts (median: 3.23 vs. 3.00 × 10^3^/µL; *p* = 0.04). C-reactive protein levels increased, and neutrophil counts decreased 8–14 days post-chemotherapy. Multivariate analysis showed that TNF > 4.7 pg/mL was inversely associated (OR = 0.27; 95% CI = 0.11–0.69; *p* = 0.0058), whereas IL-10 > 2.8 pg/mL was positively associated (OR = 3.17; 95% CI = 1.30–7.75; *p* = 0.011) with dryness. Sampling at 8–14 days post-chemotherapy predicted dryness (OR = 5.16; 95% CI = 1.32–20.09; *p* = 0.018).

**Conclusions:**

Salivary TNF and IL-10 levels and sampling timing after CT were associated with oral dryness, suggesting temporal changes in salivary inflammatory mediators.

## Introduction

Many patients with cancer develop oral mucositis (OM) during chemotherapy (CT), and the resulting inflammation and pain often lead to difficulty in oral intake and necessitate dose reduction or modification of anticancer agents [1, 2]. OM is one of the most common and debilitating complications of cancer therapy, considerably impairing patients’ quality of life and nutritional status. In severe cases, it can necessitate hospitalization or interruption of CT, thereby compromising treatment efficacy [3].

The pathophysiology of OM is multifactorial and involves a complex biological cascade initiated by CT-induced epithelial cell damage [4]. Reactive oxygen species generated by anticancer agents trigger apoptosis and necrosis in oral epithelial cells, followed by activation of nuclear factor κB and upregulation of proinflammatory cytokines, such as interleukin (IL)−6, IL-1β, and tumor necrosis factor (TNF) [5]. These mediators amplify local inflammation, recruit immune cells, and promote ulceration of the mucosal surface.

Recently, salivary biomarkers have gained attention as non-invasive tools to monitor inflammatory responses and predict OM severity. Several studies have reported increased salivary concentrations of IL-6, IL-8, TNF-α, and IL-10 during CT, suggesting their potential as predictive or diagnostic markers for OM [6, 7]. However, the role of oral environmental factors, particularly oral dryness, in modulating inflammatory responses remains poorly understood.

Oral dryness is a frequent symptom in patients undergoing CT, often resulting from reduced salivary secretion, medication effects, or mucosal inflammation [8]. This may exacerbate mucosal vulnerability by impairing lubrication and antimicrobial defense, thereby facilitating the development of OM. Despite the recognized overlap in the timing and mechanisms of oral dryness and OM [9], no study has comprehensively examined the relationship between oral dryness and salivary inflammatory mediators in patients with cancer.

Therefore, this study aimed to evaluate the association between salivary inflammatory mediators and oral dryness during oral mucositis development in patients with cancer undergoing chemotherapy. Understanding these interactions may provide insights into the pathophysiological link between salivary inflammation and mucosal toxicity, ultimately contributing to better prevention and management strategies for OM.

## Methods

### Patients

**Fig. 1:**
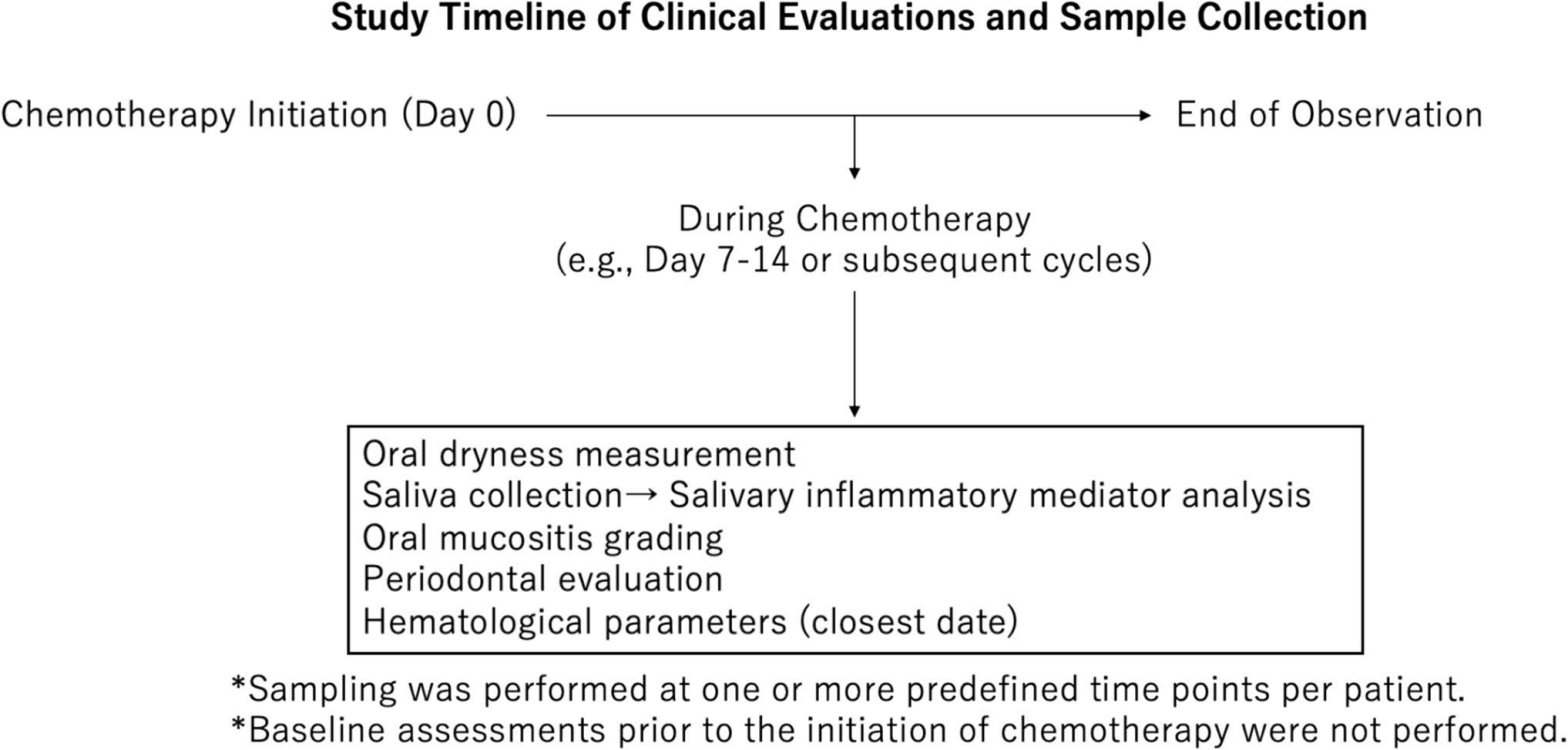
Study timeline of chemotherapy and concurrent clinical assessments. Saliva samples were collected at one or more predefined timepoints during chemotherapy and used for salivary inflammatory mediator analysis. Clinical evaluations were performed at the time of saliva collection

We enrolled patients with cancer who visited Niigata University Medical and Dental Hospital between November 2021 and August 2023 for CT or concomitant chemoradiotherapy (CCRT). At the time of intervention by the Division of Interdisciplinary Oral Management in Medical Care, OM grade was assessed, and saliva samples were collected. This study employed a time-stratified sampling approach, wherein biological samples were collected at multiple predefined timepoints following CT. Although data were obtained at several timepoints, the analysis was not designed to evaluate temporal prediction or causal relationships. The overall study timeline, including the timing of CT initiation, clinical evaluations, and saliva sample collection, is illustrated in Fig. 1.

Saliva was collected by the drooling method under standardized conditions: (i) eating and tooth brushing were completed at least 1 h before collection, (ii) patients rinsed their mouth 15 min prior to collection, and (iii) patients remained at rest for at least 5 min before collection. For patients with difficulty in secreting saliva, samples were obtained after rinsing with purified water. No active dental treatment was performed at the time of saliva collection.

We collected patient information including sex, height, weight, body mass index (BMI), cancer type, presence and grade of OM, type of anticancer drug, hematological parameters (white blood cell count, platelet count, neutrophil count, hemoglobin, C-reactive protein (CRP), total protein, and albumin), radiotherapy during the observation period, history of radiotherapy to the head and neck region, non-invasive oral care interventions within 24 h (e.g., oral rinsing, toothbrushing, interdental cleaning, sponge brushing, and oral moisturizing), presence of periodontal disease, history of hematopoietic cell transplantation, CCRT, use of OM prophylactic agents (Azunol^®^ [Nippon Shinyaku Co.,Ltd., Kyoto, Japan], *Hangeshashinto* [Tsumura & Co., Tokyo, Japan], dexamethasone 0.1% oral ointment, and povidone–iodine gargle [Meiji Seika Pharma Co., Ltd., Tokyo, Japan]), oral moisture level, and number of days after cancer therapy. These OM prophylactic agents were provided when clinically indicated as part of routine oral care and were not standardized within the study protocol. Oral moisture was measured using an oral moisture meter (Mucus^®^, THE YOSHIDA DENTAL MFG. CO., LTD, Tokyo, Japan) [10], and the median of three measurements was recorded. A score < 28 indicates oral dryness. Salivary flow rate was not assessed because it was often difficult to collect an adequate volume of saliva in patients with severe oral mucositis or pronounced oral dryness, and the procedure may impose additional burden on patients. Hematological parameters and periodontitis status were assessed at the time point closest to the saliva collection date. Blood count parameters were analyzed as continuous variables using their measured values. Periodontal disease was diagnosed by a licensed dentist based on a comprehensive clinical examination. The diagnosis was established according to the Japanese Society of Periodontology Clinical Practice Guidelines [11], including assessment of the probing pocket depth (PPD), clinical attachment loss, and gingival inflammation such as bleeding on probing. Periodontitis was defined based on these criteria, with particular consideration of sites with PPD ≥ 4 mm and/or bleeding on probing. Periodontal examinations were conducted as part of routine oral assessments performed by dental professionals during the study period. To ensure patient safety, examinations involving periodontal probing were performed only when clinically appropriate, taking into account hematological parameters and general condition. Specifically, examinations were deferred in patients with neutrophil counts < 1,000/mL, platelet counts < 50,000/mL, febrile neutropenia, grade ≥ 3 oral mucositis, grade ≥ 2 bleeding tendency, or other severe adverse events, or when deemed unsuitable by the attending physician. This approach is consistent with international recommendations to avoid invasive oral procedures during periods of severe immunosuppression. OM grade was assessed by a dentist according to the World Health Organization (WHO) oral toxicity scale (grades 0–4) [12]. Patients with OM grades 1–4 were assigned to the OM group, and those with grade 0 to the non-OM group. The timing of saliva collection was expressed as the number of days relative to the day of CT administration (day 0).

The exclusion criteria included age < 20 years; evident mucosal trauma unrelated to CT-induced OM; primary malignancy involving lesions, metastasis, or invasion of the central nervous system; participation in other clinical trials or studies; pregnancy, breastfeeding, or possible pregnancy; or other factors deemed unsuitable for study participation by the investigators.

For patient characteristics, the Wilcoxon test was used for continuous variables, and Fisher’s exact test or the chi-square test was used for categorical variables. The Mann–Whitney *U* test was used for analyses stratified by the number of days after CT. Multivariate analysis was performed using nominal logistic regression, with oral dryness (presence/absence) as the dependent variable and OM status, IL-6, IL-10, TNF, and days after CT (categorized) as independent variables. Statistical analyses were conducted using GraphPad Prism version 7 (GraphPad Software, San Diego, CA, USA) and JMP Pro version 16 (SAS Institute, Cary, NC, USA). Statistical significance was set at *p* < 0.05. The required sample size was calculated a priori based on effect size estimates obtained from our previous pilot study [6]. Assuming a two-sided significance level of 0.05 and statistical power of 80%, the minimum sample size needed to detect a clinically meaningful difference between groups was determined.

This study was approved by the Ethics Committee of Niigata University Medical and Dental Hospital (approval No. 2019-0449) and the Ethics Committee for the Research Use of Human Tissues, Tokyo University of Pharmacy and Life Sciences (approval No. 20-3). Written informed consent was obtained from all participants before sample collection and measurement.

### Samples and measurement of inflammatory mediators

Saliva accumulated in the oral cavity was collected in 2.0-mL microtubes using a saliva collection straw. Samples were stored at −20 °C until analysis. Before measurement, thawed samples were vortex-mixed and centrifuged at 3,000 rpm for 15 min at 4 °C, and the resulting supernatants were used for assays.

Salivary concentrations of IL-1β, IL-6, IL-10, IL-12p70, and TNF were quantified using the BD Cytometric Bead Array (CBA) Human Inflammation Kit (BD Biosciences, California, USA), according to the manufacturer’s instructions. Data were processed using FCAP Array™ software, version 3.0 (BD Biosciences, California, USA). Prostaglandin E2 (PGE2) and vascular endothelial growth factor (VEGF) levels were measured using enzyme-linked immunosorbent assay (ELISA) kits (Enzo Life Sciences, New York, USA; and R&D Systems, Minneapolis, MN, USA, respectively). Each assay was performed in duplicate, and mean values were used for analysis. Samples exceeding the upper detection limits (5,000 pg/mL for IL-1β, IL-6, IL-10, IL-12p70, and TNF; 2,500 pg/mL for PGE2; and 20,000 pg/mL for VEGF) were diluted up to fivefold for remeasurement. Samples that still exceeded these limits after dilution were excluded from the analysis. Salivary cytokine concentrations were categorized based on the median values observed in the OM grade 0 group (IL-1β, 196.2 pg/mL; IL-6, 30.3 pg/mL; IL-8, 1,729.4 pg/mL; IL-10, 2.8 pg/mL; IL-12p70, 1.9 pg/mL; TNF, 4.7 pg/mL; PGE2, 275.9 pg/mL; VEGF, 2,453.7 pg/mL). TNF and IL-10 were identified as inflammatory markers independently associated with oral dryness. The cutoff values for these mediators were defined as the median levels observed among patients without OM (grade 0) in the present cohort.

## Results

### Patient characteristics

A total of 162 patients were included in the analysis: 56 with oral dryness and 106 without oral dryness. Comparisons between the two groups are summarized in Table 1.

**Table 1:** Patient characteristics

		Oral dryness		
		Positive (*N* = 56)	Negative (*N* = 106)	*p* value*
Male, *n* (%)		31 (55.4)	52 (46.1)	0.45
Age^#^		61.5 (54–71)	62 (52–72)	0.76
BMI^#^ (kg/m^2^)		21.0 (19.5–24.3)	20.9 (19.2–23.9)	0.40
Cancer type, *n* (%)	Lip, oral cavity, and pharynx	4 (7.1)	8 (7.6)	0.25
	Lymphoid and hematopoietic	1 (1.8)	12 (11.3)	
	Gastrointestinal	26 (46.4)	44 (41.9)	
	Respiratory	7 (12.5)	12 (11.4)	
	Other	18 (32.1)	30 (28.3)	
History of radiotherapy to the head and neck region		2 (3.6)	3 (2.8)	0.68
Hematopoietic cell transplantation, *n* (%)		1 (1.8)	5 (4.7)	0.32
Radiotherapy during the observation period, *n* (%)		4 (7.1)	11 (10.4)	0.49
Radiation target field, *n* (%)	Head and neck	0 (0)	0 (0)	1.00
	Other regions	4 (7.1)	11 (10.4)	
Radiation dose (Gy)		33 (20–56.5)	30 (22–60)	0.69
Days after cancer treatment, *n* (%)	0–7 days	33 (62.3)	76 (74.5)	0.049*
	8–14 days	8 (15.1)	4 (3.9)	
	≥ 15 days	12 (22.6)	22 (21.6)	
Non-invasive oral care interventions within 24 h of saliva collection, *n* (%)		10 (18.5)	21 (20.4)	0.78
Prophylactic agents for OM, *n* (%)		30 (53.6)	63 (59.4)	0.47
Periodontitis, *n* (%)		35 (62.5)	52 (49.1)	0.88
OM, *n* (%)	Grade 0	36 (64.3)	62 (58.5)	0.58
	Grade 1	16 (28.6)	34 (32.1)	
	Grade 2	4 (7.1)	7 (6.6)	
	≥ Grade 3	0 (0)	3 (2.8)	
Type of anticancer drug, *n* (%)	Molecularly targeted	19 (33.9)	39 (36.8)	0.72
	Immune checkpoint inhibitors	9 (16.1)	21 (19.8)	0.56
	Hormonal therapy	1 (1.8)	1 (0.9)	0.64
	Cytotoxic	41 (73.2)	81 (76.4)	0.65
Laboratory values^#^	White blood cell count (/µL)	5415 (4220–7055)	4930 (3665–6475)	0.09
	Platelet count (×10^4^/µL)	23.8 (20.2–27.8)	24.2 (17.0–29.4)	0.48
	Neutrophil count (×10^3^/µL)	3.2 (2.3–5.1)	3.0 (2.0–4.3)	0.04*
	Hemoglobin (g/dL)	11.7 (10.7–13.1)	11.8 (10.5–12.8)	0.64
	CRP (mg/dL)	0.2 (0.06–0.7)	0.2 (0.05–0.9)	0.56
	Total protein (g/dL)	7.1 (6.6–7.7)	7.1 (6.5–7.3)	0.36
	Albumin (g/dL)	3.8 (3.4–4.1)	3.75 (3.4–4.1)	0.97
Inflammatory mediators in saliva (pg/mL)^#^	IL-1β	3.1 (0–6.6)	2.6 (0–5.7)	0.58
	IL-6	32.8 (10.1–127.5)	30.3 (10.6–118.4)	0.90
	IL-8	1760.8 (681.2–3025.7)	1558.1 (829.4–3568.5)	0.89
	IL-10	294.7 (56.7–595.7)	222.8 (79.2–553.3)	0.69
	IL-12p70	2.0 (0–3.91)	1.3 (0–4.3)	0.45
	TNF	3.5 (1.1–8.0)	4.9 (1.6–9.8)	0.25
	PGE2	327.3 (112.6–994.6)	275.0 (140.4–866.5)	0.77
	VEGF	2168.2 (1270.1–3157.7)	2156.6 (1682.9–4389.6)	0.12

^#^Median (IQR); *p < 0.05

*BMI*, body mass index; *CT*, chemotherapy; *OM*, oral mucositis; *CRP*, C-reactive protein; *IL*, interleukin; *TNF*, tumor necrosis factor; *PGE2*, prostaglandin E2; *VEGF*, vascular endothelial growth factor; *IQR*, interquartile range

No significant differences were observed in sex distribution (male: 55.4% vs. 46.1%, *p* = 0.45), age (median interquartile range [IQR] = 61.5 [54–71] vs. 62 [52–72] years, *p* = 0.76), or BMI (21.0 [19.5–24.3] vs. 20.87 [19.2–23.9] kg/m^2^, *p* = 0.40). Cancer type distribution, radiotherapy exposure, radiation target field, and radiation dose also did not differ significantly between groups (*p* = 0.25, 0.49, and 0.69, respectively). Among patients with head and neck malignancies, 5 out of 12 had a history of radiotherapy to the head and neck region prior to sample collection; however, none received radiotherapy during the hospitalization period. The history of radiotherapy to the head and neck region did not differ significantly between the oral dryness and non-dryness groups (*p* = 0.68).

The timing of sample collection relative to cancer therapy showed a significant difference (*p* = 0.049), with patients in the oral dryness–positive group more likely to have undergone sample collection within 7 days after CT (62.3% vs. 74.5%).

The proportion of patients receiving dental treatment or non-invasive oral care interventions within 24 h of saliva sampling (18.5% vs. 20.4%, *p* = 0.78), use of prophylactic agents for OM (53.6% vs. 59.4%, *p* = 0.47), prevalence of periodontitis (62.5% vs. 49.1%, *p* = 0.88), OM incidence and grade distribution (*p* = 0.58), or type of anticancer drug administered (molecularly targeted agents: 33.9% vs. 36.8%, *p* = 0.72; immune checkpoint inhibitors: 16.1% vs. 19.8%, *p* = 0.56; hormonal therapy: 1.8% vs. 0.9%, *p* = 0.64; cytotoxic agents: 73.2% vs. 76.4%, *p* = 0.65) did not differ significantly between groups. Similarly, the proportions of patients receiving radiotherapy (7.1% vs. 10.4%, *p* = 0.49) or hematopoietic cell transplantation (1.8% vs. 4.7%, *p* = 0.32) did not differ significantly between the dryness and non-dryness groups.

Regarding laboratory findings, neutrophil counts were significantly higher in the oral dryness–positive group than in the negative group (median [IQR] = 3.23 [2.34–5.07] × 10^3^/µL vs. 3.00 [2.00–4.26] × 10^3^/µL, *p* = 0.04). Other parameters including white blood cell count, platelet count, hemoglobin, CRP, total protein, and albumin levels did not differ significantly between the groups.

The concentrations of salivary inflammatory mediators such as IL-1β, IL-6, IL-8, IL-10, IL-12p70, TNF, PGE2, or VEGF did not differ significantly between the groups.

The proportions of patients with and without oral dryness were then compared (Table 2). A significant difference was observed for salivary TNF levels (*p* = 0.049), with concentrations < 4.7 pg/mL occurring more frequently in patients belonging to the oral dryness–negative group. No significant differences were observed in IL-1β, IL-6, IL-8, IL-10, IL-12p70, PGE2, or VEGF levels.

**Table 2:** Comparison of salivary inflammatory mediator concentrations between patients with and without oral dryness

		Oral dryness		
		Positive (*N* = 56)	Negative (*N* = 106)	*p* value*
IL-1β	≤ 196.2	27 (48.2%)	49 (47.6%)	0.94
	> 196.2	29 (51.8%)	54 (52.4%)	
IL-6	≤ 30.3	27 (48.2%)	52 (49.5%)	0.87
	> 30.3	29 (51.8%)	53 (50.5%)	
IL-8	≤ 1729.4	26 (47.3%)	51 (53.1%)	0.49
	> 1729.4	29 (52.7%)	45 (46.9%)	
IL-10	≤ 2.8	26 (46.4%)	57 (53.8%)	0.37
	> 2.8	30 (53.6%)	49 (46.2%)	
IL-12p70	≤ 1.9	27 (48.2%)	59 (55.7%)	0.37
	> 1.9	29 (51.8%)	47 (44.3%)	
TNF	≤ 4.7	36 (64.3%)	51 (48.1%)	0.049*
	> 4.7	20 (35.7%)	55 (51.9%)	
PGE2	≤ 275.9	26 (46.4%)	48 (50.5%)	0.78
	> 275.9	30 (53.6%)	47 (49.5%)	
VEGF	≤ 2453.7	33 (58.9%)	52 (49.5%)	0.25
	> 2453.7	23 (41.1%)	53 (50.5%)	

*IL*, interleukin; *TNF*, tumor necrosis factor; *PGE2*, prostaglandin E2; *VEGF*, vascular endothelial growth factor; *OM*, oral mucositis. All concentrations of each mediator were expressed in pg/mL

Based on the results (Table 1), which indicated a difference in the proportion of patients with oral dryness depending on the timing of saliva collection, various clinical variables were compared according to the saliva collection period (Table 3).

**Table 3:** Comparison of patient characteristics according to the number of days after cancer therapy

		0–7 days (*N* = 109)	8–14 days (*N* = 12)	≥ 15 days (*N* = 34)	*p* value*
Male, *n* (%)		54 (49.5)	8 (66.7)	16 (47.1)	0.48
Age^#^		62 (53–72)	59 (52–65.8)	65.5 (53.8–73.5)	0.37
BMI^#^ (kg/m^2^)		20.8 (19.2–23.6)	21.0 (18.8–22.9)	20.5 (18.9–24.0)	0.99
Cancer type, *n* (%)	Lip, oral cavity, and pharynx	10 (9.2)	1 (8.3)	1 (2.9)	0.48
	Lymphoid and hematopoietic	7 (6.4)	1 (8.3)	5 (14.7)	
	Other	92 (84.4)	10 (83.3)	28 (82.4)	
Radiotherapy exposure, *n* (%)		10 (9.2)	3 (25.0)	2 (5.9)	0.22
Radiation target field, *n* (%)	Head and neck	0 (0)	0 (0)	0 (0)	1.00
	Other regions	10 (9.2)	3 (25.0)	2 (5.9)	
Radiation dose (Gy)		49 (27.5–60)	30 (22–60)	11.5 (3–20)	0.18
Type of anticancer drug, *n* (%)	Cytotoxic	79 (72.5)	11 (91.7)	28 (82.4)	0.17
	Immune checkpoint inhibitors	20 (18.4)	3 (25.0)	6 (17.7)	0.85
	Hormonal therapy	1 (0.9)	0 (0)	1 (2.9)	0.61
	Molecularly targeted	40 (36.7)	5 (41.7)	11 (32.4)	0.82
Prophylactic agents for OM, *n* (%)		63 (57.8)	8 (66.7)	18 (52.9)	0.70
Periodontitis, *n* (%)		52 (91.2)	8 (100)	24 (92.3)	0.50
Non-invasive oral care interventions within 24 h of saliva collection, *n* (%)		17 (16.0)	2 (18.1)	10 (29.4)	0.25
Oral moisture		29.3 (27.5–31.2)	26.3 (25.4–30.0)	28.5 (27.5–29.9)	0.024*
Laboratory values^#^	Platelet count (×10^4^/µL)	24.4 (18.8–30)	25 (21.9–27)	22.1 (13.8–26.9)	0.15
	Hemoglobin (g/dL)	11.8 (10.9–12.9)	11.1 (10–12.7)	11.3 (9.5–12.7)	0.18
	Neutrophil count (×10^3^/µL)	3.2 (2.3–5.0)	2.8 (1.6–4.8)	2.7 (1.6–3.9)	0.026*
	Albumin (g/dL)	3.8 (3.4–4.1)	2.6 (2.6–3.8)	3.7 (3.4–4.0)	0.07
	CRP (mg/dL)	0.1 (0.05–0.7)	0.9 (0.2–2.6)	0.4 (0.1–0.9)	0.016*
	Total protein (g/dL)	7.1 (6.7–7.4)	6.7 (6.1–7.2)	6.9 (6.4–7.4)	0.13
Inflammatory mediators in saliva (pg/mL)^#^	IL-1β	196.2 (53.3–544.6)	328.4 (111.4–582.4)	230.9 (60.3–986.2)	0.68
	IL-6	22.3 (8.8–83.7)	95.6 (34.8–461.1)	57.7 (13.6–243.9)	0.04*
	IL-8	1377.8 (801.7–2905.2)	3290.3 (1727.3–5512.1)	1810.4 (621.6–2809.9)	0.054
	IL-10	2.8 (0–4.9)	2.3 (0–12.6)	2.5 (0–6.9)	0.86
	IL-12p70	2.0 (0–4.3)	0 (0–9.0)	0 (0–3.5)	0.30
	TNF	4.3 (2.3–8.7)	1.7 (0–26.6)	3.2 (1.2–10.5)	0.60
	PGE2	263.7 (114.8–769.6)	1063.7 (208.3–2003.3)	335.2 (98.6–933.8)	0.11
	VEGF	2375.4 (1533.5–4224.7)	2384.5 (931.1–3432.5)	2345.2 (1519.1–3555.8)	0.86

^#^Median (IQR); *p < 0.05

*BMI*, body mass index; *OM*, oral mucositis; *CT*, chemotherapy; *CRP*, C-reactive protein; *IL*, interleukin; *TNF*, tumor necrosis factor; *PGE2*, prostaglandin E2; *VEGF*, vascular endothelial growth factor

In the group with saliva collected on days 8–14, oral moisture levels were significantly lower and CRP levels were significantly higher than in the other groups (*p* = 0.024 and *p* = 0.016, respectively). In contrast, in the group with saliva collected on days 0–7, neutrophil counts were significantly higher than those in the other groups (*p* = 0.026).

No significant intergroup differences were evident with respect to sex, age, BMI, cancer type, dental treatment or oral care intervention status, prophylactic medication use, presence of periodontal disease, presence of OM, CT, radiotherapy exposure, radiation target field, radiation dose, hematopoietic stem cell transplantation, white blood cell count, platelet count, hemoglobin, CRP, total protein, albumin, or salivary cytokine levels.

Among salivary inflammatory mediators, IL-6 levels were significantly elevated in the 8–14-day group (*p* = 0.04). No significant differences were observed in IL-1β, IL-8, IL-10, IL-12p70, TNF, PGE2, or VEGF levels among the groups.

Based on the results of the univariate analysis, the number of days after cancer therapy and salivary concentrations of IL-6 and TNF were potentially associated with oral dryness (*p* < 0.05). In addition to these variables, IL-10 (previously reported to be associated with OM [5]) and the presence of OM were included as explanatory variables in the multivariate logistic regression analysis to evaluate their association with oral dryness (Table 4).

**Table 4:** Results of multivariate logistic regression analysis for factors associated with oral dryness

		OR [95% CI]	*p* value*
OM	None	Ref	-
	Present	0.80 [0.39–1.69]	0.57
Number of days after cancer therapy	0–7 days	Ref	-
	8–14 days	5.16 [1.32–20.09]	0.018*
	≥ 15 days	1.21 [0.51–2.86]	0.65
IL-6 (pg/mL)	≤ 30.3	Ref	-
	> 30.3	0.89 [0.41–1.92]	0.77
IL-10 (pg/mL)	≤ 2.8	Ref	-
	> 2.8	3.17 [1.30–7.75]	0.011*
TNF (pg/mL)	≤ 4.7	Ref	-
	> 4.7	0.27 [0.11–0.69]	0.0058**

*OM*, oral mucositis; *OR*, odds ratio; *CI*, confidence interval; *IL*, interleukin; *TNF*, tumor necrosis factor; *Ref.*, reference category; *CT*, cancer therapy

The multivariate analysis revealed that patients with salivary TNF concentrations > 4.7 pg/mL had significantly lower odds of oral dryness than those with TNF ≤ 4.7 pg/mL (odds ratio [OR] = 0.27, *p* = 0.0058). In contrast, patients with salivary IL-10 concentrations > 2.8 pg/mL had significantly higher odds of oral dryness than those with IL-10 ≤ 2.8 pg/mL (OR = 3.17, *p* = 0.011). Furthermore, saliva collection between 8 and 14 days after CT was significantly associated with oral dryness compared with that within 7 days (OR = 5.16, *p* = 0.018).

No significant associations were observed for the presence of OM, saliva collection at ≥ day 15, or salivary IL-6 concentrations.

## Discussion

### Principal findings

This study examined the association between salivary inflammatory mediators and oral dryness in patients with cancer undergoing CT. Multivariate analysis revealed that a salivary TNF concentration > 4.7 pg/mL was associated with approximately 0.3-fold lower odds, whereas a salivary IL-10 concentration > 2.8 pg/mL was associated with approximately 3.2-fold higher odds of oral dryness. These results suggest that TNF and IL-10 may play distinct roles in the pathophysiology of oral dryness and potentially in the onset of OM.

Salivary IL-6 levels tended to increase 8–14 days after CT, consistent with previous reports of CT-induced inflammation, although no significant relationship was observed with oral dryness. Oral dryness appeared most frequently during the same 8–14-day period, corresponding to the typical peak incidence of OM [7]. These findings highlight the importance of temporal factors in evaluating CT-related oral adverse effects.

Although neutrophil counts were significantly higher in patients with dryness than in those without, both values remained within the normal range. Therefore, this difference is unlikely to have a substantial effect on the clinical outcomes.

### Mechanistic interpretation

The inverse association between TNF and oral dryness observed in this study is noteworthy. TNF is a key proinflammatory cytokine that induces epithelial cell apoptosis and promotes mucosal inflammation [13]. However, increased TNF levels in saliva may also reflect activation of immune repair pathways or enhanced mucosal turnover, which could help preserve epithelial integrity and salivary gland function.

In contrast, IL-10, an anti-inflammatory cytokine that suppresses TNF and IL-6 signaling [14], was positively associated with oral dryness. Elevated IL-10 levels may represent a compensatory response to persistent inflammatory stress in the oral cavity or indicate epithelial and glandular dysfunction. The interplay between TNF and IL-10 likely represents a regulatory feedback mechanism that maintains mucosal homeostasis but may predispose to dysfunction when dysregulated [15]. These findings are consistent with those of prior studies linking both cytokines to oral lesions, including OM and aphthous ulcers [6, 16].

The timing of cytokine changes also warrants careful consideration. IL-6 showed an upward trend 8–14 days after CT, consistent with the known kinetics of OM development, suggesting that oral dryness and OM may share overlapping inflammatory pathways [2]. Given that most saliva samples from patients with oral dryness were collected outside this critical window, temporal variations in cytokine levels may have been underestimated.

### Clinical implications

The temporal concurrence of oral dryness and OM supports the hypothesis that both conditions share a common pathophysiological basis, namely, CT-induced oxidative stress, mucosal barrier disruption, and dysregulated cytokine signaling [5, 17–20]. Identifying this overlap offers opportunities for early intervention. Regular assessment of salivary cytokines, particularly TNF and IL-10, may enable clinicians to predict oral complications before symptom onset.

Salivary biomarkers offer a non-invasive and easily repeatable method for monitoring mucosal health. Combined with preventive oral care, saliva substitutes, or anti-inflammatory agents such as *Hangeshashinto*, which can reportedly modulate cytokine expression and alleviate CT-induced OM [21], biomarker-guided interventions could improve patient comfort, sustain nutritional intake, and prevent treatment interruptions.

Additionally, CRP levels increased 8–14 days after CT, reflecting systemic inflammatory responses, whereas neutrophil counts decreased, consistent with myelosuppression. The relatively higher neutrophil counts observed in patients with oral dryness likely reflect differences in sampling time rather than a direct association. Future synchronized sampling of saliva and blood parameters may clarify systemic local inflammatory interactions.

### Limitations and future directions

This study has some limitations. First, the findings pertaining to oral dryness were strongly influenced by the 8–14-day period. However, the number of samples in the 8–14-day group was limited (*n* = 12), which may have constrained statistical power. Therefore, further longitudinal prospective studies with an appropriately determined sample size are needed to validate these findings. Second, the sample size for certain chemotherapeutic regimens was small, limiting the ability to assess drug-specific effects. In addition, the study population included patients with a wide variety of cancer diagnoses and treatment regimens, which may differ in the onset and risk of OM. Furthermore, some patients received targeted therapies or immunotherapies, which may also have influenced the risk of oral dryness. Third, potential confounders, including radiotherapy, oral hygiene, nutritional status, mucosal microbiota, use of topical corticosteroids (e.g., dexamethasone ointment), administration of targeted therapies or immune checkpoint inhibitors, and the presence of periodontitis, were not fully controlled, although some of these factors were partially assessed. Fourth, owing to the time-stratified sampling design, the temporal and causal relationships between oral dryness and inflammatory markers could not be determined. Therefore, it is unclear whether the changes in inflammatory markers precede the development of or occur as a consequence of oral dryness. In addition, the observed associations may be influenced by unmeasured confounding factors. Finally, baseline oral assessments and saliva sampling prior to the initiation of CT were not performed. Therefore, the pretreatment oral inflammatory status of the patients could not be determined, and the observed changes during CT should be interpreted with caution. In addition, this study did not include objective measurement of salivary flow rate, which may provide a more direct assessment of hyposalivation. In patients with severe oral mucositis, however, collection of sufficient saliva or use of conventional methods such as the gauze technique may be difficult or may increase patient burden and risk of mucosal injury.

Future studies should adopt longitudinal, multi-timepoint sampling combined with standardized OM grading and patient-reported outcomes for oral dryness. Such approaches could clarify causal pathways linking inflammatory mediators to mucosal toxicity. Integration of salivary cytokine monitoring into oncology practice may ultimately facilitate early identification of at-risk patients and guide personalized strategies for oral complication management.

## Conclusion

In summary, salivary TNF and IL-10 concentrations, along with the timing of sampling after CT, were associated with oral dryness in patients with cancer. These results suggest that temporal changes in salivary inflammatory mediators may be related to oral dryness during CT. However, further studies are required to clarify the relationships between salivary inflammatory mediators, oral dryness, and OM.

## Data Availability

No datasets were generated or analysed during the current study.

## References

[1] Cinausero M, Aprile G, Ermacora P et al (2017) New frontiers in the pathobiology and treatment of cancer regimen-related mucosal injury. Front Pharmacol 8:354. 10.3389/fphar.2017.00354 28642709 10.3389/fphar.2017.00354 PMC5462992

[2] Brown TJ, Gupta A (2020) Management of cancer therapy-associated oral mucositis. JCO Oncol Pract 16(3):103–109. 10.1200/JOP.19.00652 32048926 10.1200/JOP.19.00652

[3] Baskar K, Patil A, Ramya K, Yadav TB (2025) A comprehensive review of oral mucositis and its management. Cross Current Int J Med Biosci 7(2):9–23. 10.36344/ccijmb.2025.v07i02.002

[4] Miyano K, Kono T, Uezono Y (2015) A challenge to overcome stomatitis of cancer patients treated with chemotherapy. Folia Pharmacol Jpn 146(2):76–80. 10.1254/fpj.146.76 10.1254/fpj.146.76 26256744

[5] Sonis ST (2009) Mucositis: the impact, biology and therapeutic opportunities of oral mucositis. Oral Oncol 45(12):1015–1020. 10.1016/j.oraloncology.2009.08.006 19828360 10.1016/j.oraloncology.2009.08.006

[6] Kiyomi A, Yoshida K, Arai C et al (2022) Salivary inflammatory mediators as biomarkers for oral mucositis and oral mucosal dryness in cancer patients: A pilot study. PLoS One 17(4):e0267092. 10.1371/journal.pone.0267092 35476641 10.1371/journal.pone.0267092 PMC9045655

[7] Yoshida K, Kiyomi A, Kurokawa A et al (2024) Association between salivary inflammatory mediators and oral mucositis in patients with cancer undergoing chemotherapy. Support Care Cancer 32(9):625 39222245 10.1007/s00520-024-08836-1

[8] Hosseini MS, Sanaie S, Mahmoodpoor A et al (2024) Cancer treatment-related xerostomia: basics, therapeutics, and future perspectives. Eur J Med Res 29(1):571. 10.1186/s40001-024-02167-x 39614391 10.1186/s40001-024-02167-x PMC11607820

[9] Walsh M, Fagan N, Davies A (2023) Xerostomia in patients with advanced cancer: a scoping review of clinical features and complications. BMC Palliat Care 22(1):178. 10.1186/s12904-023-01276-4 37950188 10.1186/s12904-023-01276-4 PMC10638744

[10] Life Co., Ltd. (Tokyo, Japan). https://life-qol.net/

[11] The Japanese Society of Periodontology Clinical Practice Guidelines for the Periodontal Treatment 2022. https://www.perio.jp/publication/upload_file/guideline_perio_2022_en.pdf

[12] World Health Organization. WHO handbook for reporting results of cancer treatment. World Health Organization; 1979. https://iris.who.int/server/api/core/bitstreams/c1b590fa-20a6-4c97-9123-5cc4d34c7cdb/content

[13] Lykowska-Szuber L, Walczak M, Skrzyczak M et al (2021) Effect of anti-TNF therapy on mucosal apoptosis genes expression in Crohn’s disease. Front Immunol 12:615539. 10.3389/fimmu.2021.615539 33767696 10.3389/fimmu.2021.615539 PMC7985326

[14] Donnelly RP, Freeman SL, Hayes MP (1995) Inhibition of IL-10 expression by IFN-γ up-regulates transcription of TNF-α in human monocytes. J Immunol 155(3):1420–1427. 10.4049/jimmunol.155.3.1420 7636207

[15] Ouyang W, O’Garra A (2019) IL-10 family cytokines IL-10 and IL-22: from basic science to clinical translation. Immunity 50(4):871–891. 10.1016/j.immuni.2019.03.020 30995504 10.1016/j.immuni.2019.03.020

[16] Krzaczek MP, Mitura-Lesiuk M, Zawitkowska J et al (2019) Salivary and serum concentrations of selected pro- and antiinflammatory cytokines in relation to oral lesions among children undergoing maintenance therapy of acute lymphoblastic leukemia. Contemp Oncol 23(2):81–86. 10.5114/wo.2019.85878 10.5114/wo.2019.85878 PMC6630390 31316289

[17] Lalla RV, Bowen J, Barasch A et al (2014) MASCC/ISOO clinical practice guidelines for the management of mucositis secondary to cancer therapy. Cancer 120(10):1453–1461. 10.1002/cncr.28592 24615748 10.1002/cncr.28592 PMC4164022

[18] Hong CHL, Gueiros LA, Fulton JS et al (2019) Systematic review of basic oral care for the management of oral mucositis in cancer patients and clinical practice guidelines. Support Care Cancer 27(10):3949–3967. 10.1007/s00520-019-04848-4 31286232 10.1007/s00520-019-04848-4

[19] Hitomi S, Ujihara I, Sago-Ito M et al (2019) Hyposalivation due to chemotherapy exacerbates oral ulcerative mucositis and delays its healing. Arch Oral Biol 105:20–26. 10.1016/j.archoralbio.2019.06.003 31238198 10.1016/j.archoralbio.2019.06.003

[20] Scully C, Sonis S, Diz PD (2006) Oral mucositis. Oral Dis 12(3):229–241. 10.1111/j.1601-0825.2006.01258.x 16700732 10.1111/j.1601-0825.2006.01258.x

[21] Miyano K, Eto M, Hitomi S et al (2020) The Japanese herbal medicine Hangeshashinto enhances oral keratinocyte migration to facilitate healing of chemotherapy-induced oral ulcerative mucositis. Sci Rep 10(1):625. 10.1038/s41598-019-57192-2 31953420 10.1038/s41598-019-57192-2 PMC6969174

